# Global survey on the utilisation and experiences with different retrobulbar anaesthesia techniques in horses

**DOI:** 10.1111/evj.70082

**Published:** 2025-08-23

**Authors:** Simona Lieberth, Katharina Thieme, Christian Dancker, Roswitha Merle, Johanna Corinna Eule

**Affiliations:** ^1^ Unit for Ophthalmology, Centre for Veterinary Clinical Services School of Veterinary Medicine, Freie Universität Berlin Berlin Germany; ^2^ Equine Clinic, Veterinary Hospital School of Veterinary Medicine, Freie Universität Berlin Berlin Germany; ^3^ Valera Small Animal Clinic Berlin Berlin Germany; ^4^ Institute of Veterinary Epidemiology and Biostatistics, School of Veterinary Medicine, Freie Universität Berlin Berlin Germany

**Keywords:** enucleation, horse, nerve block, ocular, orbit

## Abstract

**Background:**

Retrobulbar anaesthesia (RBA) is relevant for ocular surgery in standing sedated horses.

**Objectives:**

Gathering insights on experiences with RBA techniques.

**Study Design:**

Cross‐sectional survey.

**Methods:**

An online survey collected information on the respondents' professional background, indications, injection methods, use of ultrasound assistance, medication, and complications associated with RBA in horses.

**Results:**

Two‐hundred and thirty‐eight veterinarians from 25 countries contributed. 86.1% were members of veterinary specialty colleges. RBA indications were enucleation (97.8%), corneal surgery (29.5%), eyelid surgery, paracentesis of the vitreous and anterior chamber, cataract surgery and vitrectomy (<10.0% each). The dorsal approach was most commonly used (88.8%), followed by the 4‐point (34.8%), lateral (8.9%), and modified Peterson blocks (2.2%). Ultrasound‐assisted needle positioning was used by 16.1%. Most commonly administered drugs were mepivacaine (67.4%), lidocaine (56.3%), and bupivacaine (37.9%). Complications included lack of anaesthesia (40.6%), exophthalmos (33.5%), chemosis (32.1%), and retrobulbar haemorrhage (22.8%). The choice of injection method and reported complications were significantly influenced by participants' professional backgrounds: as horse caseload percentage increased, dorsal injection use decreased (*p* = 0.011, OR 0.981, 95% CI 0.967–0.996), injection‐related complications increased (*p* < 0.001, OR 1.015, 95% CI 1.006–1.024), and postoperative complications decreased (*p* < 0.001, OR 0.983, 95% CI 0.976–0.991). As ophthalmic case percentage increased, dorsal injection use increased (*p* = 0.009, OR 1.022, 95% CI 1.006–1.039), 4‐point injection method decreased (*p* = 0.044, OR 0.993, 95% CI: 0.986–1.000), injection‐related complications decreased (*p* < 0.001, OR 0.985, 95% CI 0.978–0.993), and postoperative complications increased (*p* < 0.001, OR 1.019, 95% CI 1.012–1.027).

**Main Limitations:**

Results may reflect selection and recall bias; complication prevalence was not assessed.

**Conclusions:**

Dorsal and 4‐point blocks are the most used, varying by participants' professional background. RBA causes mostly mild complications; severe ones are rare.

## Resumen

**Historial:**

La anestesia retrobulbar (RBA), es relevante en las cirugías oculares en el caballos sedado de pie.

**Objetivos:**

Acumular información de las experiencias con técnicas de RBA.

**Diseño del Estudio:**

Encuesta transversal.

**Métodos:**

Una encuesta en línea recolecto información sobre la formación profesional de los entrevistados, las indicaciones, métodos de inyección, uso de asistencia con ultrasonido, drogas y complicaciones asociadas con RBA en caballos.

**Resultados:**

238 veterinarios de 25 países contribuyeron. 86.1% pertenecían de un colegio veterinario de especialización. Las indicaciones para RBA eran enucleaciones (97.8%), cirugía corneal (29.5%), cirugía de parpado, paracentesis del vitreo y cámara anterior, cirugía de catarata y vitrectomía (<10.0% cada una). El abordaje dorsal fue el más comúnmente usado (88.8%), seguido por el de 4 puntos (34.8%), lateral (8.9%), y bloqueo de Peterson modificado (2.2%). Posicionamiento de la aguja asistido por ultrasonido fue usado por 16.1%. Los fármacos usados más comúnmente fueron mepivacaina (67.4%), lidocaina (56.3%), bupivacaina (37.9%). Las complicaciones incluyeron falta de anestesia (40.6%), exoftalmos (33.5%), quemosis (32.1%), y hemorragia retrobulbar (22.8%). La elección del método de inyección y las complicaciones fueron significativamente influenciadas por la formación profesional de los participantes: a medida que el porcentaje de casos equinos aumento, las inyecciones dorsales disminuyeron (*p* = 0.011, OR 0.981, 95% CI 0.967–0.996), las complicaciones relacionadas a las inyecciones aumentaron (*p* < 0.001, OR 1.015, 95% CI 1.006–1.024), y las complicaciones post operativas disminuyeron (*p* < 0.001, OR 0.983, 95% CI 0.976–0.991). A medida que el porcentaje de casos oftalmológicos aumento, el uso de inyección dorsal aumento (*p* = 0.009, OR 1.022, 95% CI 1.006–1.039), el método de inyección en 4 puntos disminuyo (*p* = 0.044, OR 0.993, 95% CI: 0.986–1.000), las complicaciones relacionada a inyecciones disminuyeron (*p* < 0.001, OR 0.985, 95% CI 0.978–0.993), y las complicaciones post operativas aumentaron (*p* < 0.001, OR 1.019, 95% CI 1.012–1.027).

**Limitaciones Principales:**

Los resultados pueden reflejar la selección y el sesgo de memoria: la prevalencia de las complicaciones no fue evaluada.

**Conclusiones:**

Los bloqueos dorsal y en 4‐puntos fueron los más utilizados, dependiendo de la formación profesional de cada participante. RBA causa en su mayoría complicaciones leves: las severas son raras.

## 1 INTRODUCTION

Surgical procedures on sedated horses require effective pain management, with local anaesthesia playing a key role in reliably blocking nociceptive signal transmission.^1^ The development of enhanced sedation and local anaesthetic protocols has enabled the effective bypassing of the risks associated with general anaesthesia, a matter which continues to represent a significant concern in equine patients.^2^ The mortality rate for healthy horses undergoing general anaesthesia is approximately 0.9%.^2^ Furthermore, ophthalmic procedures are associated with a higher risk of complications compared to selected non‐ophthalmic surgeries.^3^ The eye and its adnexa are innervated by sensory, motor, and autonomic nerve fibres, all of which must be considered when planning ophthalmic surgery. The most relevant nerves, except the auriculopalpebral nerve, are located within the retrobulbar muscle cone. This cone‐shaped space is bordered by the globe, the extraocular muscles and their fascia. It contains cranial nerves (CN) II along with its surrounding meninges, III, IV, V, and VI, the ciliary ganglion, blood and lymph vessels, and retrobulbar fat. The optic nerve (CN II) enters the orbit via the optic canal, while the motor nerves (CN III, IV, and VI) access through the orbital fissure to innervate the extraocular muscles. The ophthalmic branch of the trigeminal nerve (CN V) enters through the orbital fissure along with the motor nerves. It provides sensory innervation to the eye and adnexa by dividing into the supraorbital, lacrimal, and infratrochlear nerves. The maxillary branch of CN V enters through the alar foramen and provides sensory innervation to the lower eyelid via the zygomatic nerve.^4^ These nerves can either be blocked individually or together using retrobulbar anaesthesia (RBA). The auriculopalpebral nerve, a motoric branch of the facial nerve (CN VII), innervates the orbicularis oculi muscle for eyelid closure and runs externally along the zygomatic process^4^ and must be blocked separately, as it cannot be targeted via an RBA.

Four techniques for RBA in horses have been described: the dorsal,^5^ lateral^6^ and modified Peterson block,^7^ all aim to deposit local anaesthetics inside the retrobulbar muscle cone. The fourth technique, the 4‐point block^5^ aims to create four deposits of local anaesthetics behind the globe's equator. Local anaesthetics include lidocaine, mepivacaine, ropivacaine, and bupivacaine, with injection volumes ranging from 4 to 40 mL.^5^, ^6^, ^7^, ^8^ Mixtures of lidocaine and mepivacaine (1:1)^9^ or bupivacaine (1:1)^10^ have also been reported. Ultrasound‐guided needle placement is becoming more common in human and veterinary ophthalmology for peripheral nerve blocks,^11^ and equine cadaveric studies have shown potential benefits in improving precision and reducing complications.^12^, ^13^, ^14^

No previous studies have examined current clinical practice regarding RBA. Therefore, this study aimed to gain knowledge through an online survey to compare techniques and complications observed in routine practice with those described in the literature and to explore the impact of professional characteristics and nerve block techniques on complication rates. Additionally, the participants' local anaesthetic protocols during the enucleation procedures in standing, sedated patients were investigated.

## 2 MATERIALS AND METHODS

### 2.1 Questionnaire design and distribution

A cross‐sectional online survey (Survey S1) was conducted using the LimeSurvey open‐source platform (Cloud version 5.6.43). Topics covered included indications, injection techniques, preferred methods, drugs used, ultrasound assistance, and associated complications for RBA as well as standard local anaesthetic approaches to enucleation in standing sedated patients. Additionally, respondents were asked about their professional background.

The survey primarily included multiple‐choice questions (MCQ, only one answer can be selected) and multiple answer questions (MAQ, more than one answer may be selected). All questions included an option to provide alternative written responses. LimeSurvey's logic functions were used to skip non‐applicable questions based on participants' responses. The content and structure of the questionnaire were reviewed by diplomates of the European College of Veterinary Ophthalmologists (ECVO) and European College of Veterinary Anaesthesia and Analgesia (ECVAA). A diplomate of the European College of Veterinary Public Health with specific expertise in statistics and epidemiology contributed to its statistical design.

The survey was accessible online for 6 months (May–November 2023) and was designed to be completed within approximately 10 min. The survey was distributed via flyers at various conferences, including the 2023 Annual Scientific Meetings of the ECVO, the European College of Veterinary Surgeons (ECVS), the International Equine Ophthalmology Consortium (IEOC), and the 31st Bavarian Veterinary Congress. Additionally, it was shared through mailing lists of the ECVS, the ECVAA, the American College of Veterinary Anaesthesia and Analgesia (ACVAA), the American College of Veterinary Ophthalmologists (ACVO), the German Association of Equine Veterinary Practitioners (GPM), the equine veterinary mailing list of Freie Universität Berlin, and was also announced in the print newsletter of the German Veterinary Journal (issue 08/2023).

Participation was voluntary, anonymous, and restricted to one response per IP address.

Participants were required to accept the privacy policy before proceeding.

### 2.2 Data analysis

Data were exported from LimeSurvey and recorded in Microsoft Excel (version 2022; Microsoft Corp.). Statistical analyses were performed using IBM SPSS Statistics (version 29.0; IBM Corp.). For data visualisation, R (version 4.4.0; R Core Team, 2024) within RStudio (version 2025.5.0.496; RStudio Team, 2025) was used, utilising the ggplot2 package (version 3.5.1; Wickham, 2024).

Inclusion criteria were involvement in RBA (indicated by an affirmative response to the first question) and complete information on professional background. Exclusion criteria included no experience with RBA and incomplete professional background information. Due to the non‐mandatory structure of the survey, skipped questions were treated as missing data, resulting in varying sample sizes (N) (Table S1). Therefore, throughout the manuscript, different sample sizes (*N*) occur. Alternative answers were treated as predefined options and included in the results unless they appeared only once, in which case they were removed from analysis. For analysis of the injection volumes, the upper value of the range was used when a range was reported. This adjustment reflected the respondents' indication of using lower volumes for ponies and higher volumes for horses. Values with ‘+/−’ notation were recorded as the stated number without the ‘+/−’. Throughout the manuscript, MAQ answers are indicated in brackets; if unspecified, the question was a MCQ.

Descriptive analyses included frequencies and cross‐tabulations for demographic information, indications for RBA, use of anaesthetics, block techniques, ultrasound assistance, complications, and local anaesthetic protocols for enucleation in standing sedated patients. For inferential data analysis, statistical significance was set at *p* < 0.05. Binary logistic regression (reporting regression coefficients [b], odds ratios [OR], and 95% confidence intervals [CI]) and Fisher's exact test (including OR and 95% CI) were used to evaluate the effect of years of experience on the probability of a complication and the impact of the proportion of horses and ophthalmic cases treated on the choice of injection method and/or the probability of reporting complications.

#### 2.2.1 Analysis of complications reported for different injection methods

In order to be able to attribute complications to a specific injection method, answers of respondents who used multiple techniques were excluded for this analysis. A Fisher's exact test, including OR and 95% CI, was used to assess the association between injection method and reported complications.

#### 2.2.2 Analysis of impact of professional characteristics

Two subsets of respondents were evaluated based on specific professional profile criteria for a detailed evaluation of indications, injection methods, and complications. The *ophthalmology group* (*n* = 65) consisted of members of the American/European College of Veterinary Ophthalmologists (A/ECVO) and/or national specialists/trainees in ophthalmology whose equine patients were exclusively ophthalmic patients. The *equine group* (*n* = 120) comprised members of the American/European College of Veterinary Surgeons (A/ECVS), American/European College of Veterinary Anaesthesia and Analgesia (A/ECVAA), and/or national specialists/trainees in veterinary surgery, anaesthesia, or horses, whose equine ophthalmology cases accounted for up to 20% of their overall caseload. The remaining respondents (*n* = 53) did not meet the inclusion criteria for either subgroup and were excluded from this specific analysis.

The two subgroups were compared using frequencies to explore differences in indications, injection techniques, and complications. Additionally, Fisher's exact tests were performed to compare injection techniques, the use of ultrasound guidance, and the reported complication rates between groups.

## 3 RESULTS

**FIGURE 1:**
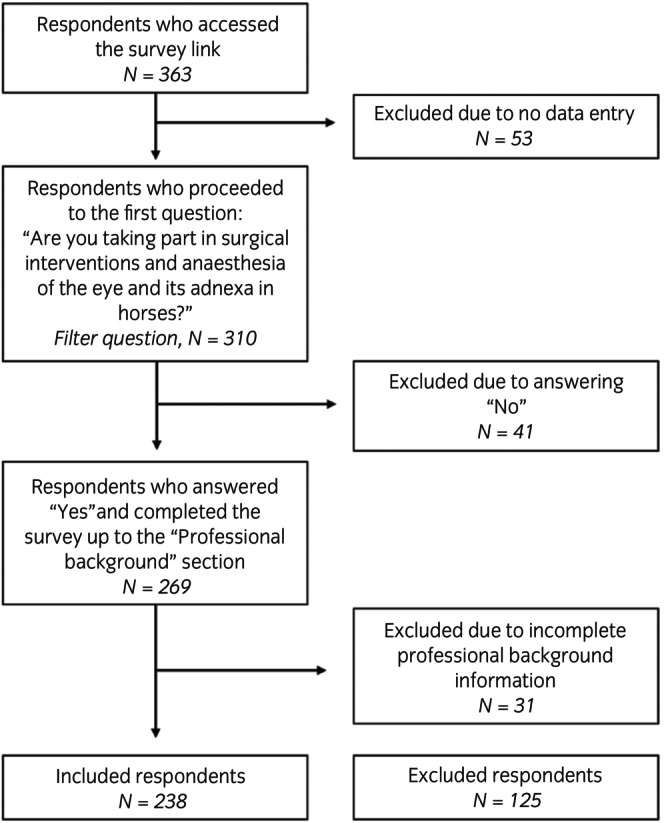
Flowchart illustrating the exclusion process of data sets from an online questionnaire on retrobulbar anaesthesia in horses based on the predefined exclusion criteria: ‘no experience with RBA’ and ‘incomplete professional background information’.

Of the 363 respondents, 238 clinicians were included. Exclusions (*n* = 125) were due to missing data (*n* = 53), no experience with RBA (*n* = 41), or incomplete professional background information (*n* = 31) as outlined in Figure 1.

### 3.1 Descriptive data analysis

#### 3.1.1 Demographic information

**FIGURE 2:**
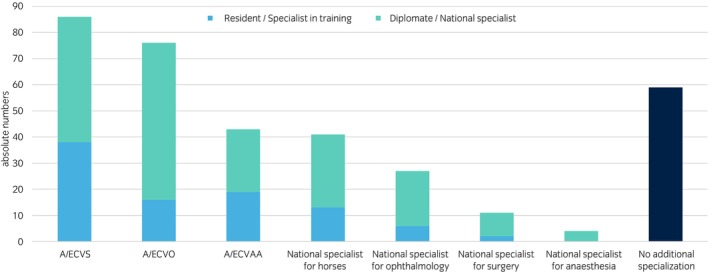
Bar chart illustrating participants' qualifications in absolute numbers (*N* = 238, multiple answer question, more than one answer may be selected). A/ECVAA, American/European College of Veterinary Anaesthesia and Analgesia; A/ECVO, American/European College of Veterinary Ophthalmologists; A/ECVS, American/European College of Veterinary Surgeons.

The study included 238 veterinarians from 25 countries across six continents (Table S2). Most respondents practiced in Europe (70.2%, 167/238) or North America (23.1%, 55/238). By country, the highest representation was observed in Germany (22.3%, 53/238), the United States (20.6%, 49/238), and the United Kingdom (14.7%, 35/238). 86.1% (205/238) of participants were members of veterinary specialty colleges (Figure 2). More than half (53.4%, 127/238) had over 10 years of equine practice experience, and half (50%, 119/238) managed equine patients in 90%–100% of their cases.

#### 3.1.2 Use of RBA

Participants primarily used RBA (MAQ) for enucleation (97.8%, 219/224), with lower frequencies for corneal surgery (29.5%, 66/224), eyelid surgery (9.8%, 22/224), paracentesis of the vitreous (9.4%, 21/224), paracentesis of the anterior chamber (8.5%, 19/224), cataract surgery (7.6%, 17/224) and vitrectomy (2.7%, 6/224). The RBA was performed in hospital settings by 70.3% of the respondents (154/219), in field settings by 6.3% (15/219), and in both settings by 22.8% (50/219). All procedures were performed under standing sedation as well as general anaesthesia (Table S1).

#### 3.1.3 Use of local anaesthetics

Mepivacaine was the most commonly used anaesthetic (67.4%, 151/224, MAQ), with 27.2% of the respondents (61/224) using it exclusively. Lidocaine (56.3%, 126/224) and bupivacaine (37.9%, 85/224) were the second and third most popular choices, with 11.6% (26/224) and 4.9% (11/224) of the participants using them exclusively, respectively. Ropivacaine (3.1%, 7/224) and procaine (0.9%, 2/224) were rarely administered. Adrenaline was added by 6.5% (14/217) of the respondents.

A total of 20.5% (45/219) reported combining different local anaesthetics in a single syringe (Table S3).

#### 3.1.4 Use of block technique

The dorsal block was the most commonly used and preferred block technique, followed by the 4‐point block. In contrast, the lateral and modified Peterson blocks were rarely used (Table 1). Five respondents indicated that their preferred techniques varied depending on the specific procedure. The most frequently used needle size was 20G across all block techniques. Additional details on the needle size and length are provided in Tables S4 and S5.

**TABLE 1:** Injection methods for retrobulbar anaesthesia used (*N* = 224) and preferred (*N* = 216) by participants of an online survey, presented as absolute numbers (*n*) and percentages (%), grouped by injection technique.

Injection technique	Used technique MAQ		Preferred technique MCQ	
	*n* = 224	%	*n* = 216	%
Dorsal block	199	88.8	172	79.6
4‐point block	78	34.8	40	18.5
Lateral block	20	8.9	2	0.9
Modified Peterson block	5	2.2	1	0.5

Abbreviations: MAQ, multiple answer questions (more than one answer may be selected). MCQ, multiple choice question (only one answer may be selected).

For the 4‐point block, 75.3% (58/77) used a curved needle, 16.9% (13/77) used a straight needle, and 7.8% (6/77) used both. Median injection volumes ranged from 10 to 20 mL (Table S6). The median injection volume for the 4‐point block was double that for the dorsal block (Tables S6 and 2).

**TABLE 2:** Comparison of injection volume in millilitre used for retrobulbar anaesthesia between the dorsal and 4‐point block techniques; only respondents using a single technique included.

Injection technique	*N*	Median	Minimum	Maximum	IQR 25	IQR 75
Dorsal block	133	10.0	5.0	25.0	10.0	10.0
4‐point block	13	20.0	6.0	40.0	10.0	20.0

Abbreviation: IQR, inter quartile range.

#### 3.1.5 Use of ultrasound assistance

The use of ultrasound for needle positioning was analysed for dorsal, lateral, and modified Peterson blocks. Overall, 16.1% (36/224) of the respondents used ultrasound assistance for needle positioning, whereas 83.9% (188/224) did not. Of those using ultrasound assistance, 61.1% (22/36) used it in up to 50% of cases, while 38.9% (14/36) used it in more than 50% of cases. Divided by the block technique, ultrasound assistance was used by 16.6% (33/199) of the respondents applying the dorsal block and 15% (3/20) of the respondents applying the lateral block. None of the participants performing the modified Peterson block (0/5) used ultrasound assistance.

#### 3.1.6 Reported complications

Reported complications related to RBA (MAQ) were analysed based on the time of observation: during injection, intraoperatively, and postoperatively (*N* = 224). During the injection, 31.3% of the respondents reported complications (70/224). The reported complications included retrobulbar haemorrhage (22.8%, 51/224), oculocardiac reflex (5.4%, 12/224), globe puncture (4.9%, 11/224), intravascular injection (3.6%, 8/224), and optic nerve puncture (3.1%, 7/224). No cases of intrameningeal injection were observed. Intraoperative complications were reported by 75.4% (169/224) of practitioners, with the most common being lack of anaesthesia (40.6%, 91/224), exophthalmos (33.5%, 75/224), and chemosis (32.1%, 72/224). Other reported issues included surgical field compression due to local anaesthetic (17.4%, 39/224), lack of akinesia (16.1%, 36/224), hypersensitivity reactions (3.1%, 7/224), and oculocardiac reflex (2.1%, 5/224).

Postoperatively, 27.7% (62/224) of the participants reported complications. These included chemosis (19.2%, 43/224), exophthalmos (8.9%, 20/224), keratitis (3.6%, 8/224), and retrobulbar abscesses (3.1%, 7/224).

Regardless of the timing, 46.8% (101/216) of the respondents reported failed RBAs. The majority of these respondents (72.3%, 73/101) encountered failure in up to 30% of cases, whereas 27.7% (28/101) reported failure in more than 30% of cases. Strategies to deal with the failure of anaesthesia included repeated application of the RBA in 82.2% (83/101) and/or subcutaneous infiltration of the surgical field in 62.4% (63/101) of the cases. When the RBA was reapplied, 51.8% (43/83) used the same injection method, while 48.2% (40/83) opted for a different technique.

#### 3.1.7 Enucleation in the standing sedated patient

For enucleation in standing sedated patients (MAQ), 89.0% (186/209) of participants utilised an RBA. 86.1% (180/209) employed an auriculopalpebral nerve block. The most common approach was a combination of RBA, auriculopalpebral, supraorbital, lacrimal, infratrochlear, and zygomatic nerve blocks, along with topical local anaesthetic on the cornea (21.5%, 45/209). A total of 6.2% (13/209) of the participants used a combination of RBA, auriculopalpebral nerve block, and upper and lower lid infiltrations. Four respondents used a combination of RBA and auriculopalpebral nerve block exclusively. The remaining 72.7% (152/209) employed 62 different unique nerve block combinations.

Twenty‐three individuals did not use RBA in the standing sedated patient (Table S7).

### 3.2 Inferential data analysis

#### 3.2.1 Influence of years of experience on complications

Of the 224 participants who answered the questions on complications, 185 (82.6%) had more than 5 years of work experience. At all three time points, the group with more than 5 years of professional experience reported higher complication rates than did the group with less than 5 years of experience; however, these differences were not statistically significant: 33.5% versus 20.5% during injection (*p* = 0.130, OR 1.953, 95% CI: 0.847–4.502), 76.7% versus 69.2% intraoperatively (*p* = 0.3, OR 1.468, 95% CI: 0.686–3.141), and 28.1% versus 25.6% postoperatively (*p* = 0.9, OR 1.134, 95% CI: 0.516–2.491). Longer work experience increased the likelihood of experiencing a failed RBA at least once in one's career. Participants with more than 15 years of work experience had twice the odds of reporting failed RBA compared to those with less than 2 years of work experience, although this was not statistically significant (*p* = 0.4, OR 2.151, 95% CI: 0.394–11.748).

Some rare complications were reported exclusively by participants with more than 5 years of experience; although these findings were not significant. The oculocardiac reflex was observed by 12/185 participants during injection and 5/185 intraoperatively. Postoperative abscesses were reported by 7/185 participants. None of the 39 participants with less than 5 years of experience reported observing these complications.

#### 3.2.2 Influence of proportion of horses and equine ophthalmic cases in the caseload

**FIGURE 3:**
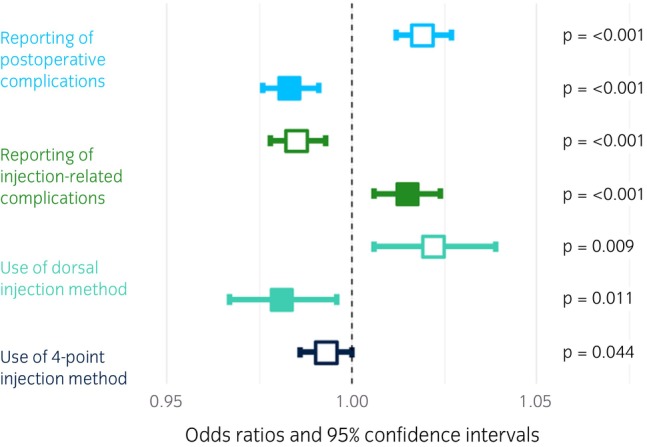
Forest plot illustrating the odds ratios and 95% confidence intervals for the effects of the proportions of horses and ophthalmic cases treated by the responding veterinarians on the choice of injection method and the reporting of complications. The effect of the proportion of horses in the caseload is represented by solid squares, while the effect of the proportion of ophthalmic cases is indicated by open squares.

The logistic regression model revealed a significant influence of the proportion of horses in a participant's caseload on both the choice of injection method and the observation of complications (Figure 3). As the percentage of horses in the caseload increased, the use of the dorsal injection method decreased (b = −0.019, *p* = 0.011, OR 0.981, 95% CI: 0.967–0.996), the observation of injection‐related complications increased (b = 0.015, *p* < 0.001, OR 1.015, 95% CI: 1.006–1.024), and the reporting of postoperative complications decreased (b = − 0.017, *p* < 0.001, OR 0.983, 95% CI: 0.976–0.991).

Furthermore, the logistic regression model revealed a significant influence of the proportion of ophthalmic cases in the participants' equine caseload on both the choice of injection method and the observation of complications (Figure 3). As the percentage of ophthalmic cases increased, the use of the dorsal injection method increased (b = 0.022, *p* = 0.009, OR 1.022, 95% CI: 1.006–1.039), while the use of the 4‐point injection method decreased (b = −0.007, *p* = 0.044, OR 0.993, 95% CI: 0.986–1.000), the observation of injection‐related complications decreased (b = −0.015, *p* < 0.001, OR 0.985, 95% CI: 0.978–0.993), and the reporting of postoperative complications increased (b = 0.019, *p* < 0.001, OR 1.019, 95% CI: 1.012–1.027).

#### 3.2.3 Influence of block technique on complications

Of the 238 respondents, 156 reported using a single injection technique for RBA exclusively. These answers were used to assess the influence of the injection technique on complications (see Section 3.2.1). The dorsal block was used exclusively by 137/156 respondents, the 4‐point block by 18/156, and the lateral block by 1/156. The lateral block was excluded from further analyses because of insufficient data.

All complications were compared between the dorsal and 4‐point techniques, but no significant differences were observed apart from ‘lack of anaesthesia’. This complication was reported intraoperatively by 41.6% (57/137) of the respondents using the dorsal block and 16.7% (3/18) using the 4‐point block, making it 3.6 times more often with the dorsal than with the 4‐point block (*p* = 0.041, OR 3.563, 95% CI: 0.985–12.881).

#### 3.2.4 Comparison between the *ophthalmology* and the *equine group*

The *ophthalmology group* represented 27.3% (65/238) of the participants, whereas the *equine group* accounted for 50.4% (120/238). The remaining 22.4% (53/238) were excluded from the following analysis (see Section 3.2.2).

##### Indications

**FIGURE 4:**
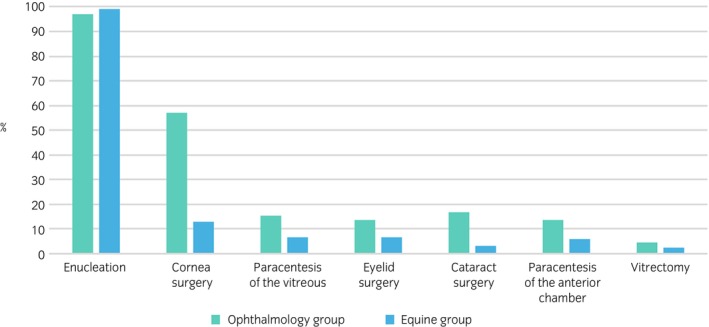
Bar chart illustrating percentage distribution of surgical indications based on the comparison between the *ophthalmology* and *equine groups*. Multiple answer question, more than one answer may be selected. O*phthalmology group*: Members of the A/ECVO and/or national specialists/trainees in ophthalmology, whose equine patients were exclusively ophthalmic patients; *N* = 65. *Equine group*: Members of the A/ECVS, A/ECVAA and/or national specialists/trainees in veterinary surgery, anaesthesia, or horses, whose equine ophthalmology cases accounted for up to 20% of their overall caseload; *N* = 120. A/ECVAA, American/European College of Veterinary Anaesthesia and Analgesia; A/ECVO, American/European College of Veterinary Ophthalmologists; A/ECVS, American/European College of Veterinary Surgeons.

Indications for RBA differed between groups, as detailed (Figure 4). *The ophthalmology group* performed globe‐sparing procedures 2.7 times more frequently than the *equine* group did.

##### Injection technique

Both groups primarily used the dorsal block over the 4‐point block, although the *equine group* showed a greater preference for the 4‐point block than did the *ophthalmology group* (39.2% vs. 26.2%, *p* = 0.150, OR 1.818, 95% CI: 0.936–3.534). When performing the 4‐point block, the median injection volume was 20 mL in the *equine group* compared to 40 mL in the *ophthalmology group*.

Ultrasound assistance for needle positioning was used by 23.4% (28/120) of the *equine group* and 4.6% (3/63) of the *ophthalmology group*, reflecting a 6.3 times greater usage in the *equine group* (*p* < 0.001, OR 6.290, 95% CI: 1.832–21.593).

##### Reported complications

During injection, the *equine group* reported more complications than did the *ophthalmology group* (42.5% vs. 13.8%), predominantly retrobulbar haemorrhage (32.5% vs. 6.2%) (Table 3).

**TABLE 3:** Reported complication rates based on the comparison between the *ophthalmology* and *equine subgroups*, grouped by time point of observation.

Timepoint	Complication	*Ophthalmology* group, *N* = 65	%	*Equine* group, *N* = 120	%	*p*	OR	95% CI
During injection	Overall	9	13.8	51	42.5	<0.001	4.60	2.08–10.15
	Retrobulbar haemorrhage	4	6.2	39	32.5	<0.001	7.34	2.49–21.65
	Oculocardiac reflex	4	6.2	7	5.8	>0.999	1.06	0.29–3.75
	Globe puncture	2	3.1	8	6.7	0.498	2.25	0.46–10.92
	Intravascular injection	0	0.0	7	5.8	0.098	1.06	1.02–1.11
	Optic nerve puncture	0	0.0	6	5.0	0.092	1.05	1.01–1.10
	Intrameningeal injection	0	0.0	0	0.00	n.a	n.a	n.a
Intraoperatively	Chemosis	40	61.5	15	12.5	<0.001	11.24	5.38–23.26
	Lack of anaesthesia	21	32.3	61	50.8	0.020	2.17	1.15–4.07
	Lack of akinesia	18	27.7	13	10.8	0.007	3.15	1.43–6.94
	Overall	53	81.5	91	75.8	0.459	1.41	0.66–2.94
	Exophthalmos	20	30.8	42	35.0	0.626	1.21	0.64–2.31
	Surgical field compression	8	12.3	25	20.8	0.165	1.88	0.79–4.44
	Hypersensitivity	2	3.1	3	2.5	>0.999	1.23	0.20–7.69
	Oculocardiac reflex	1	1.5	4	3.3	0.658	2.21	0.24–20.17
Postoperatively	Overall	33	50.8	20	16.7	<0.001	5.15	2.60–10.20
	Chemosis	28	43.1	8	6.7	<0.001	10.64	4.45–25.00
	Exophthalmos	9	13.8	9	7.5	0.196	1.96	0.75–5.26
	Keratitis	2	3.1	3	2.5	>0.999	1.23	0.20–7.69
	Retrobulbar abscess	2	3.1	4	3.3	>0.999	1.09	0.19–6.10

*Note:* O*phthalmology group*: members of the A/ECVO and/or national specialists/trainees in ophthalmology whose equine patients were exclusively ophthalmic patients. *Equine group*: members of the A/ECVS, A/ECVAA and/or national specialists/trainees in veterinary surgery, anaesthesia, or horses, whose equine ophthalmology cases accounted for up to 20% of their overall caseload.

Abbreviations: A/ECVAA, American/European College of Veterinary Anaesthesia and Analgesia; A/ECVO, American/European College of Veterinary Ophthalmologists; A/ECVS, American/European College of Veterinary Surgeons; CI, confidence interval; n.a, not applicable; OR, odds ratio; *p*, *p*‐value.

Intraoperatively, the *ophthalmology group* observed higher rates of lack of akinesia (27.7% vs. 10.8%) and chemosis (61.5% vs. 12.5%), while the *equine group* more frequently reported a lack of anaesthesia (50.8% vs. 32.3%). Postoperatively, the *ophthalmology group* reported more complications than did the *ophthalmology group* (50.8% vs. 16.7%), with chemosis being the most common (43.1% vs. 6.7%). No factors beyond the reported findings were identified as influencing the complications. The choice of drugs and the length or size of the cannula had no impact.

## 4 DISCUSSION

The RBA is a nerve block technique widely used by practitioners across various disciplines worldwide for a broad range of indications. To our knowledge, this is the first study to provide data on the clinical utilisation rates of RBA in equine patients. Enucleation was the most common indication for RBA, which aligns with expectations since complications related to globe injury are no longer a concern in these cases. Notably, all reported procedures were performed under standing sedation and general anaesthesia, a finding that was particularly unexpected for vitrectomy (*n* = 2) and cataract surgery (*n* = 1) (Table S1).

The number of RBA‐associated complications reported increased with greater participant work experience; although this finding was not statistically significant. This finding likely reflects the fact that the data were collected based on individual clinicians' experiences rather than on the number of cases. More experienced clinicians may have encountered and documented a broader range of complications over time, rather than complications increasing per procedure. In addition, their clinical expertise may aid in the early recognition of subtle changes and potentially contribute to the identification of these complications.

### 4.1 *Ophthalmology* versus *equine group*

To investigate the preferences for RBA techniques and associated complications based on the participants' areas of expertise, two subgroups were analysed (*ophthalmology* and *equine groups*). Both groups mainly used the dorsal block, but the *ophthalmology group* favoured it over the 4‐point block more than the *equine group* did. This difference may be attributed to variations in the standard reference literature and indications for RBA procedures. The foundational textbook for equine surgery, *Auer* et al.'s *Equine Surgery*,^15^ and the standard reference for equine ophthalmology, *Gilger's Equine Ophthalmology*,^16^ provide different guidance. Both texts caution against the 4‐point block, noting that it may increase intraocular pressure and the risk of corneal perforation. Notably, *Gilger's Equine Ophthalmology* ^16^ specifically advises against the 4‐point block for intraocular surgeries, emphasising its lack of advantages over the dorsal block. In combination with the higher reporting of globe‐sparing surgeries within the *ophthalmology group*, this likely contributed to the observed differences in technique preferences between the groups.

### 4.2 Complications general and *ophthalmology* versus *equine group*

The reported complications align with those described in the literature.^15^, ^16^, ^17^ Notably, complications in our study were recorded at the clinician level rather than per case, which may influence the interpretation of complication rates by reflecting individual recall rather than procedural frequency. Frequently reported complications were generally mild, whereas severe complications were less commonly observed. Severe complications were exclusively reported by participants with more than 5 years of professional experience. Intrameningeal injection—a serious complication previously documented in a cat^18^—was not reported in this study. This absence is particularly noteworthy, as an intrameningeal injection during a standing procedure could result in catastrophic outcomes for the horse and pose significant risks to the attending staff. The oculocardiac reflex, causing hypotonia and bradycardia from globe manipulation or traction, was detected during both general anaesthesia and sedation. However, a detection bias toward general anaesthesia is likely due to the differing monitoring protocols between general anaesthesia and standing sedation, potentially leading to underreporting in the latter. Additionally, differential diagnoses, including electrolyte imbalances, anaphylactic reactions to drugs, and underlying cardiac diseases, should be considered.

The observation of inadequate intraoperative anaesthesia varied significantly depending on the injection technique used. The 4‐point block was associated with higher success in achieving adequate anaesthesia than was the dorsal block; however, the reported volume of local anaesthetic used for the 4‐point block was double that used for the dorsal block (Table 2). It remains unclear whether the improved anaesthetic effect was attributable to the technique itself or to the increased anaesthetic volume. Larger anaesthetic volumes may lead to complications, such as exophthalmos, chemosis, obstruction of the surgical field, and increased intraocular pressure. Although these complications are of limited concern in enucleation, they can complicate or impede other surgical procedures.^15^, ^16^ Furthermore, opioid use during procedures was not inquired and could have potentially influenced these results.

When comparing complications between the groups, the *ophthalmology group* reported a higher observation of chemosis, both intraoperatively and postoperatively, than the *equine group*. This likely reflects their greater focus on globe‐sparing surgery.

The higher rate of missing akinesia noted by the *ophthalmology group* is consistent with the critical importance of globe immobility in globe sparing procedures. Lower perception of akinesia in the *equine group* may be attributed to the fact that fewer globe sparing procedures are done and that in enucleation, akinesia can only be effectively assessed at the start of surgery.

In contrast, the *equine group* reported a higher rate of insufficient anaesthesia than the *ophthalmology group*. Two factors may explain this observation. First, the median anaesthetic volume used in the *equine group* was lower than that in the *ophthalmology group*. Second, the *ophthalmology group's* specialised training in ocular procedures likely increased the number of cases and thereby enhanced their proficiency in performing RBAs, which may have contributed to better anaesthesia outcomes.

When discussing the complications associated with RBA, it is crucial to acknowledge that they may not be solely attributable to the RBA itself, as it is often used in combination with other local nerve blocks. Furthermore, most research on RBA has focused on enucleation procedures, limiting the significance of the findings regarding potential globe damage. *McKinney* ^17^ analysed various RBA procedures and found that the complication rates were generally low. However, three of the five studies included in the analysis combined RBA with other local nerve blocks.^8^, ^19^, ^20^ Despite this limitation, complications were reported in 8/251 RBA procedures, resulting in a relatively low overall complication rate of 3.2%.

When evaluating only the two studies^7^, ^21^ that exclusively utilised RBA, the only complication reported among the 17 procedures was bradycardia (5.9%).

Another clinical study reported a single case of chemosis in 15 dorsal RBAs performed without additional nerve blocks (6.7%).^22^

### 4.3 Enucleation in the standing sedated patient

In our study, a wide range of anaesthetic techniques was reported for enucleation in standing sedated patients. This has led to insufficient case numbers for individual combinations and their associated complications, hindering robust statistical analysis.

From an anatomical perspective, the combination of a RBA and an auriculopalpebral nerve block is expected to provide adequate anaesthesia for enucleation in a standing sedated patient. All relevant sensory nerves emerge from the posterior aspect of the bony orbit.^4^ Blocking the auriculopalpebral nerve provides motoric blockade of the orbicularis oculi muscle, thereby facilitating surgery. However, the observation that most participants used additional nerve blocks prompts further investigation.

A possible explanation could be lack of confidence in the RBA technique. Moreover, a mobile eyelid, attributable to an insufficient auriculopalpebral nerve block, could be misinterpreted for inadequate analgesia. Therefore, implementing additional nerve blocks at the outset may reduce the risk of requiring supplemental blocks later in the procedure, which could otherwise prolong the surgical duration. Reducing procedural time is critical for lowering the risk of surgical site infections, tissue trauma,^23^ and patient sedation time. In equine patients, concerns about the amount of local anaesthetics are typically negligible owing to their limited clinical impact in larger species,^24^ which further reinforces this approach.

Another explanation could be the differing interpretations of best practices, potentially shaped by published protocols that advocate combining RBA and auriculopalpebral nerve block with additional local blocks for enucleation.^4^, ^8^, ^9^, ^10^, ^15^, ^19^

Notably, 23 participants did not use RBA for enucleation in standing patients. While alternative approaches are possible, achieving sufficient anaesthesia without RBA poses a challenge and may fall short of meeting optimal animal welfare standards.

### 4.4 Use of drugs

No correlation was identified between any single anaesthetic drug and the reported complications, indicating that all drugs reported were similarly safe for horses undergoing RBA. Notably, none of the anaesthetic agents were associated with chemosis or hypersensitivity reactions previously linked to lidocaine.^5^ Consequently, the choice of local anaesthetic should primarily focus on its onset and duration of action. Lidocaine and mepivacaine are fast onset intermediate‐acting agents, whereas bupivacaine is a slow onset long‐acting agent.^1^ Owing to its prolonged duration of action, bupivacaine is particularly well suited for extended or highly painful procedures; yet start of surgery has to be adapted to its delayed onset of around 20 min.

Approximately one‐fifth of the participants reported the use of mixtures of anaesthetic drugs in a single syringe. Studies have shown that a combination of anaesthetic agents does not preserve or enhance their individual beneficial properties. For example, combining a fast onset, short duration agent with a slow onset, long duration agent does not yield a combination of rapid onset and prolonged duration. Instead, the resulting anaesthetic properties can be unpredictable due to factors such as the pH of the anaesthetic solution. Additional alterations in tissue pH due to disease may further influence the onset and duration of mixed anaesthetics, potentially compromising their effectiveness and amplifying their toxic properties.^1^

### 4.5 Popularity of injection techniques

Based on our data, the modified Peterson block appears to be infrequently utilised, and its application is not widely documented in original research articles, aside from the initial description by *Raffe* et al. in 1986,^7^ where the technique was adapted from its use in cattle. It remains referenced in some textbooks as a potential method for RBA^15^, ^16^ but is not recommended by *Gilger's Equine Ophthalmology*.^16^ *McKinney* ^17^ highlighted the challenge of accurately determining the exact location of local anaesthetic deposition using this method.

Similar concerns extend to the lateral RBA, which has not been described in original studies evaluating RBAs in equines.^17^ Although the lateral approach was mentioned in a tutorial article by *Labelle & Clark‐Price*,^6^ it has not been incorporated into the previously cited standard literature.^15^, ^16^

The limited inclusion of both techniques in foundational and peer‐reviewed equine literature, coupled with insufficient data on their success rates and complications, underscores their restricted practicality and applicability in equine clinical practice.

In contrast, dorsal and 4‐point blocks are well‐documented in the literature, both in standard references^15^, ^16^, ^25^ and original research articles.^8^, ^19^, ^20^, ^21^, ^26^, ^27^ This extensive documentation is reflected in the relatively high usage rates observed in our dataset. The greater availability of literature on these techniques also contributes to a broader understanding of the potential complications. For instance, a study by *Gilger and Davidson* ^20^ involving 189 horses undergoing dorsal blocks, of which 80%–85% were globe‐sparing procedures, reported only two complications, highlighting a relatively low‐risk profile. Given the rarity of complication reports on globe‐sparing procedures using RBA, this study provides reassuring evidence for the safety of the dorsal block.^20^

### 4.6 Ultrasonography and future studies

Although most of the observed complications are not life‐threatening, minimising them represents an important goal for improving animal welfare. Notably, the finding that nearly one‐third of the participants (27.7%, 28/101) in our study reported failed RBA in over 30% of cases underscores the need to enhance the reliability of the block. Several studies have evaluated the impact of ultrasound assistance on RBA in equine cadavers. *Morath* et al.^12^ and *Thieme* et al.^14^ evaluated this for the dorsal block, while *Leigh* et al.^13^ investigated a peribulbar approach. Both studies^13^, ^14^ comparing ultrasound to blind injection demonstrated that ultrasound improved needle placement at the intended site. These encouraging results suggest that ultrasound can aid in needle positioning; however, their validity is limited as they were not conducted on live animals. *Tooley* et al.^22^ recently conducted a clinical study on a low‐dose (5 mL) dorsal RBA using ultrasound guidance for needle placement. They demonstrated that a low volume administered at a precise location can yield successful outcomes. However, they did not achieve satisfactory lid desensitisation.^22^

The findings of these studies collectively suggest that ultrasound can significantly improve the success rate of RBA. The usage rates for ultrasound assistance reported in our study (16.6% for the dorsal approach and 15.0% for the lateral approach) suggest that it is not widely used. Ultrasound can be easily integrated into the routine application of RBA as a non‐invasive and readily available tool in equine practice. However, further research on that topic is required, especially in clinical settings.

### 4.7 Limitations

The survey distribution method and in‐ and exclusion criteria may have introduced selection bias, reflecting only a subset of equine veterinarians. The exclusion of 31 respondents due to incomplete professional background information may introduce some bias. However, their responses were reviewed prior to exclusion and did not indicate any distinct patterns or associations with a specific subgroup. Therefore, we do not consider this a substantial source of bias. Furthermore, data collection at the clinician level rather than at the horse level hindered the ability to determine the actual frequency of observed complications. This limitation should be interpreted in the context of the low complication rates reported in other RBA studies. Additionally, exploratory data analyses, which focused on subsets of respondents, reduced the sample size and, consequently, the statistical power. Finally, we acknowledge that the accuracy of the information provided by participants cannot be independently verified and that recall bias may have influenced the responses.

## 5 CONCLUSION

RBA is a widely used nerve block technique in equine practice and is generally associated with mild side effects. Severe, life‐threatening complications are rare, underscoring RBA's relative safety. The dorsal and 4‐point block techniques were the most employed approaches overall, with varying degrees of utilisation depending on the respondents' professional backgrounds. Participants with predominantly ophthalmological expertise favoured the dorsal approach, consistent with the recommendations of the ophthalmological textbooks for globe‐sparing surgeries. Based on this survey, there was no standardised anaesthetic approach for enucleation in standing sedated patients, as reflected by the wide variety of nerve block combinations employed. A large‐scale, multicentre study could systematically evaluate RBA procedures, including ultrasound‐guided approaches, through a comparative and prospective assessment of complication rates. This would help identify best practices and optimise procedural protocols.

## FUNDING INFORMATION

No funding.

## CONFLICT OF INTEREST STATEMENT

The authors have declared no conflicting interests.

## AUTHOR CONTRIBUTIONS

**Simona Lieberth:** Conceptualization; investigation; data curation; formal analysis; methodology; validation; funding acquisition; visualization; project administration; writing – original draft. **Katharina Thieme:** Conceptualization; investigation; validation; visualization; writing – review and editing; supervision. **Christian Dancker:** Writing – review and editing. **Roswitha Merle:** Formal analysis; methodology; validation; visualization; writing – review and editing. **Johanna Corinna Eule:** Conceptualization; investigation; validation; visualization; supervision; resources; funding acquisition; writing – review and editing.

## DATA INTEGRITY STATEMENT

Simona Lieberth had full access to all the data in the study and takes responsibility for the integrity of the data and the accuracy of the data analysis.

## ETHICAL ANIMAL RESEARCH

The study was approved by the Central Ethics Committee of Freie Universität Berlin (ZEA‐Nr. 2024‐002).

## INFORMED CONSENT

By completing the questionnaire, clinicians consented to participation in the study.

## Supporting information


**Data S1.** Survey S1: Questionnaire used for cross‐sectional online survey.

**Table S1:** Information collected on respondents and details associated with retrobulbar anaesthesia in horses and the use of local anaesthetic techniques.

**Table S2:** Countries where respondents were employed at the time of completing the survey.

**Table S3:** Mixture of anaesthetics drugs used by respondents.

**Table S4:** Details on needle size used by respondents.

**Table S5:** Details on needle length used by respondents.

**Table S6:** Volume of local anaesthetic used by respondents.

**Table S7:** Local anaesthetic approach used for enucleation in standing horses by 23 participants who did not use retrobulbar anaesthesia.

## Data Availability

The data that support the findings of this study are openly available in ‘figshare’ at http://doi.org/10.6084/m9.figshare.29944694.
