# Supplementary material for: Global survey on the utilisation and experiences with different retrobulbar anaesthesia techniques in horses
Source: Equine Vet J. 2025 Aug 23;58(4):1091–102. doi: 10.1111/evj.70082 (PMC13244178; doi:10.1111/evj.70082)
Supplement: Supplementary file 1 — Data S1. Survey S1: Questionnaire used for cross‐sectional online survey. [file EVJ-58-1091-s004.pdf]

**Survey S1:** Questionnaire used for a cross-sectional online survey among equine veterinarians on the use of retrobulbar anaesthesia.

## COVER LETTER

Dear colleagues!
We would like to share our survey about **retrobulbar anaesthesia in horses** with you and would be very pleased about your responses.
The questionnaire can be filled out conveniently via computer or mobile phone.
**Link:** https://vetepi.limesurvey.net/898325?lang=en
Please find the **QR Code** attached to this mail.
Deadline for this survey is November 30th 2023.

Feel free to contact s.lieberth@fu-berlin.de in case you have any concerns or feedback.
We are looking forward to your replies!

With best wishes from Berlin,
S. Lieberth, Dr. K. Thieme and Prof. C. Eule

## SURVEY

**Privacy policy**
To continue please first accept our survey data policy.
Privacy policy: I consent to my data being processed, stored and used for the purpose of scientific research questions related to this survey.

**Abbreviations**
MCQ, multiple choice question, only one answer can be selected
MAQ, multiple answer question, more than one answer may be selected

**Retrobulbar anaesthesia – general questions**

In this section we would like to get an overview about your usage of retrobulbar anaesthesia.

Q1

MCQ

Are you taking part in surgical interventions and anaesthesia of the eye and its adnexa in horses? Feel free to use the comment box, in case you want to add any information.

O yes

O no

-> if no, forwarded to "personal information"

Q2

In which surgical procedures are you taking part? Please specify, whether the horse is in general anaesthesia or in standing sedation.

| | Enucleation | Eyelid surgery | Cornea surgery | Paracentesis of anterior chamber | Paracentesis of vitreous body | Cataract surgery | Vitrectomy |
|---|---|---|---|---|---|---|---|
| Standing sedation | O | O | O | O | O | O | O |
| General anaesthesia | O | O | O | O | O | O | O |
| Both | O | O | O | O | O | O | O |
| Not performing | O | O | O | O | O | O | O |
| No answer | O | O | O | O | O | O | O |

Q3

MCQ

Are you using retrobulbar anaesthesia? Feel free to use the comment box, in case you want to add any information.

O yes
O no

-> if no, please share some insights, why you are not using retrobulbar anaesthesia in the comment box.

Q4

MAQ

For which procedures are you using retrobulbar anaesthesia?

| | |
|---|---|
| | O Enucleation<br><br>O Eyelid surgery<br><br>O Cornea surgery<br><br>O Paracentesis of anterior chamber<br><br>O Paracentesis of vitreous body<br><br>O Cataract surgery<br><br>O Vitrectomy<br><br>O Other: ________ |
| Q5<br><br>**MAQ** | Please estimate the overall percentage of your patients being sedated vs being in general anaesthesia while performing retrobulbar anaesthesia.<br><br>O Sedated in ___ %<br><br>O General anaesthesia in ___ % |

| | |
|---|---|
| Q6<br><br>**MAQ** | Under which conditions are you applying retrobulbar anaesthesia?<br><br>O Clinic conditions<br><br>O Field conditions<br><br>O Other: ________ |
| Q7<br><br>**MCQ** | Are you experiencing the situation, that your initial retrobulbar block is not showing the desired effect?<br><br>O no<br><br>O yes in ___ %<br><br>-> if yes, which methods do you use to deal with the situation? **MAQ**<br><br>O Subcutaneous infiltration of the surgical field<br><br>O Reapply retrobulbar block in the same fashion as the original block<br><br>O Reapply retrobulbar block in another fashion |

| | |
|---|---|
| | O Other: ________ |
| Q8<br><br>MAQ | Which local anaesthetics are you routinely using for retrobulbar anaesthesia?<br><br>Please estimate their proportional percentage.<br><br>O Bupivacaine in ___ %<br><br>O Lidocaine in ___ %<br><br>O Mepivacaine in ___ %<br><br>O Procaine in ___ %<br><br>O Ropivacaine in ___ %<br><br>O Other: ________ in ___ % |
| Q9<br><br>MCQ | Are you mixing different local anaesthetics within one syringe?<br><br>O no<br><br>O yes -> if yes, please indicate the used anaesthetics and ratio. If your combination is not listed below, please indicate the used anaesthetics and ratio (MAQ)<br><br>O Lidocaine : Bupivacaine ratio 1:2<br><br>O Lidocaine : Mepivacaine ratio 1:2<br><br>O Lidocaine : Mepivacaine ratio 1:1<br><br>O Lidocaine : Bupivacaine ratio 1:1<br><br>O Other ratio ________ : ________ ratio _ : _ |
| Q10<br><br>MCQ | Are you combining adrenaline (vasoconstrictive effect) with local anaesthetic?<br><br>O no<br><br>O yes in ___ % |
| Q11<br><br>MAQ | Which complications are you observing during injection of retrobulbar anaesthesia?<br><br>O None<br><br>O Globe puncture<br><br>O Intrameningeal injection<br><br>O Intravascular injection |

| | |
|---|---|
| | O Oculocardiac reflex<br>O Optic nerve puncture<br>O Retrobulbar haemorrhage<br>O Other: ________ |

| | |
|---|---|
| Q12<br>**MAQ** | Which complications are you observing during surgery after use of retrobulbar anaesthesia?<br><br>O None<br>O Chemosis<br>O Compromised surgical field due to tissue swelling<br>O Exophthalmos<br>O Hypersensitivity reaction due to used medication (Urticaria, strong retrobulbar swelling)<br>O Lack of akinesia<br>O Lack of anaesthesia<br>O Oculocardiac reflex<br>O Other: ________ |
| Q13<br>**MAQ** | Which complications are you observing postoperatively after use of retrobulbar anaesthesia?<br><br>O None<br>O Chemosis<br>O Exophthalmos<br>O Exposition keratitis<br>O Retrobulbar abscess or cellulitis<br>O Other: ________ |

**Retrobulbar anaesthesia – technique specific questions**

In this section we would like to collect specific information about retrobulbar anaesthesia.

| Q14<br><br>MAQ | Which injection techniques are you using for retrobulbar anaesthesia? To ensure that everybody is on the same page, the blocks will be briefly described.<br><br>**O 4-point-block:** needles are placed transconjunctival close to the orbital rim at four positions deep into the orbit aiming to inject the anaesthetic into the retrobulbar space.<br><br>**O Dorsal block:** a straight needle is inserted into the supraorbital fossa, just caudal to the dorsal orbital rim.<br><br>**O Lateral block:** a straight needle is placed cranial to the ramus of the mandible and ventral to the ventral border of the zygomatic bone, just ventral to the facial crest.<br><br>**O Modified Peterson block:** a curved needle is placed 1cm lateral to the lateral canthus and then advanced ventromedial into the orbit. |
|---|---|
| Q15<br><br>MCQ | Which injection technique are you preferring? Feel free to use the comment box, in case you want to add any information.<br><br>O 4-point-block<br><br>O Dorsal block<br><br>O Lateral block<br><br>O Modified Peterson block |

| Q16 | Dorsal technique |
|---|---|
| A | For the dorsal approach, which injection volume are you using in millilitre?<br><br>___ ml |
| B<br><br>MAQ | For the dorsal approach, which size of cannula are you using? 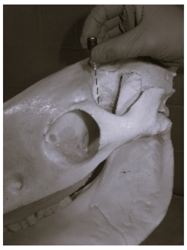<br><br>O 18G<br><br>O 19G<br><br>O 20G<br><br>O 21G<br><br>O 22G<br><br>O I don’t know<br><br>O Other: ________ |

| | |
|---|---|
| C<br><br>**MAQ** | For the dorsal approach, which length of cannula are you using?<br><br><br>O 2,5“= 63mm<br><br>O 3“= 75mm<br><br>O 3,5“= 90mm<br><br>O I don’t know<br><br>O Other: ________ |
| D | In how many percent of your dorsal retrobulbar blocks are you using ultrasound assistance?<br><br>____ % |

| Q17 | Lateral block |
|---|---|
| A | For the lateral approach, which injection volume are you using in millilitre?<br><br>____ ml |
| B<br><br>**MAQ** | For the lateral approach, which size of cannula are you using?<br><br>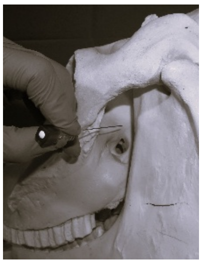<br><br>O 18G<br><br>O 19G<br><br>O 20G<br><br>O 21G<br><br>O 22G<br><br>O I don’t know<br><br>O Other: ________ |
| C<br><br>**MAQ** | For the lateral approach, which length of cannula are you using?<br><br><br>O 2,5“= 63mm<br><br>O 3“= 75mm<br><br>O 3,5“= 90mm<br><br>O I don’t know<br><br>O Other: ________ |

| | |
|---|---|
| D | In how many percent of your lateral retrobulbar blocks are you using ultrasound assistance?<br><br>___ % |

| | |
|---|---|
| Q18 | Modified Peterson block |
| A | For the modified Peterson block, which injection volume are you using in millilitre?<br><br>___ ml |
| B<br><br>**MAQ** | For the modified Peterson block, which size of cannula are you using? 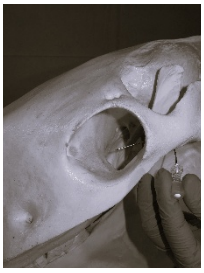<br><br>O 18G<br><br>O 19G<br><br>O 20G<br><br>O 21G<br><br>O 22G<br><br>O I don't know<br><br>O Other: ________ |
| C<br><br>**MAQ** | For the modified Peterson block, which length of cannula are you using?<br><br>O 2,5"= 63mm<br><br>O 3"= 75mm<br><br>O 3,5"= 90mm<br><br>O I don't know<br><br>O Other: ________ |
| D | In how many percent of your modified Peterson blocks are you using ultrasound assistance?<br><br>___ % |

| | |
|---|---|
| Q19 | 4-point-block |

| | |
|---|---|
| A | For the 4-point-block, which injection volume are you using in total in millilitre?<br><br>___ ml |
| B<br><br>MAQ | For the 4-point-block, which size of cannula are you using?<br><br>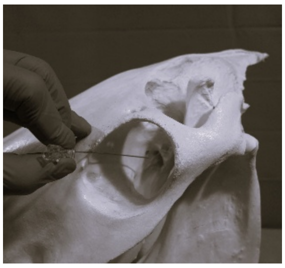<br><br>O 18G<br><br>O 19G<br><br>O 20G<br><br>O 21G<br><br>O 22G<br><br>O I don’t know<br><br>O Other: ________ |
| C<br><br>MAQ | For the 4-point-block, which length of cannula are you using?<br><br>O 2,5“= 63mm<br><br>O 3“= 75mm<br><br>O 3,5“= 90mm<br><br>O I don’t know<br><br>O Other: ________ |
| D | Are you using a straight or curved cannula for the 4-point-block? Feel free to use the comment box, in case you want to add any information.<br><br>O Straight<br><br>O Curved<br><br>O Both |

## Enucleation

| | |
|---|---|
| Q20<br><br>MAQ | What is your standard local anaesthetic approach for enucleation in the standing sedated horse? |

| | |
|---|---|
| | O None<br>O Auriculopalpebral nerve **AP**<br>O Infiltration anaesthesia in the lower lid<br>O Infiltration anaesthesia in the upper lid<br>O Infratrochlear nerve **I**<br>O Lacrimal nerve **L**<br>O Local anaesthesia of the cornea<br>O Retrobulbar anaesthesia<br>O Supraorbital nerve **S**<br>O Zygomatic nerve **Z**<br>Feel free to use the comment box, in case you want to add any information. 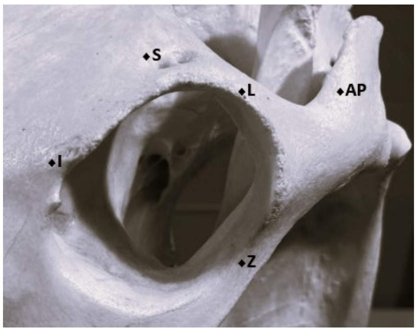  |

## Personal info

In this section we would like to collect some information about your clinical background.

| | |
|---|---|
| Q21<br>**MAQ** | Which of the following qualifications do you have?<br><br>O None<br>O Additional designation / sub-specialty „ophthalmology“<br>O Diplomate ECVAA / ACVAA<br>O Diplomate ECVO / ACVO<br>O Diplomate ECVS / ACVS<br>O Veterinary specialist for anaesthesia (national)<br>O Veterinary specialist for horses (national)<br>O Veterinary specialist for surgery (national) |
| Q22<br>**MAQ** | Are you currently taking part in any training programme?<br><br>O No<br>O Additional designation / sub-specialty „ophthalmology“<br>O Resident ECVAA / ACVAA<br>O Resident ECVO / ACVO<br>O Resident ECVS / ACVS |

| | |
|---|---|
| | O Veterinary specialisation for anaesthesia<br><br>O Veterinary specialisation for horses<br><br>O Veterinary specialisation for surgery |
| Q23<br><br>**MCQ** | How long have you been working in the equine practical field?<br><br>O < 2 years<br><br>O 2 -5 years<br><br>O 6 – 10 years<br><br>O 11 - 15 years<br><br>O > 15 years |
| Q24 | What percentage of your patients are horses?<br><br>____ % |
| Q25 | What percentage of your horse patients are ophthalmological patients?<br><br>____ % |
| Q26 | In which country are you currently working? |
