# Supplementary material for: Global survey on the utilisation and experiences with different retrobulbar anaesthesia techniques in horses
Source: Equine Vet J. 2025 Aug 23;58(4):1091–102. doi: 10.1111/evj.70082 (PMC13244178; doi:10.1111/evj.70082)
Supplement: Supplementary file 2 — Table S1: Information collected on respondents and details associated with retrobulbar anaesthesia in horses and the use of local anaesthetic techniques. [file EVJ-58-1091-s007.pdf]

Equine Veterinary Journal BEVA

Table S1: Information was collected on the professional characteristics of the respondents (238 equine clinicians), as well as indications, clinical setting, use of drugs, used and preferred injection methods and complications associated with retrobulbar anaesthesia (RBA) in horses. Additionally, the use of local anaesthetic techniques for enucleation in the standing sedated horse was assessed.
Data are presented as numbers (n/N) and percentages (%).

**Variable**	**%**	**n/N**
Years in equine practice (MCQ)		
< 2	3.4	8/238
2 - 5	15.1	36/238
6 - 10	28.2	67/238
11 - 15	17.2	41/238
> 15	36.1	86/238
% of equines in patient caseload		
0 - 50	42.4	101/238
51 - 99	20.6	49/238
100	37	88/238
% of ophthalmic cases in equine patient caseload		
< 20	58.8	140/238
21 - 99	10.9	26/238
100	30.3	72/238
Indications for RBA (MAQ)		
Enucleation	97.8	219/224
Cornea surgery	29.5	66/224
Eyelid surgery	9.8	22/224
Paracentesis of vitreous	9.4	21/224
Paracentesis of anterior chamber	8.5	19/224
Cataract surgery	7.6	17/224
Vitrectomy	2.7	6/224
Indications for surgery, grouped by the anaesthetic state of the patient (MAQ)		
Enucleation		
Sedation	48.4	109/225
General anaesthesia	6.2	14/225
Both	45.3	102/225
Eyelid surgery		
Sedation	40.4	84/208
General anaesthesia	6.7	14/208
Both	52.9	110/208
Cornea surgery		
Sedation	12.5	21/168
General anaesthesia	29.8	50/168
Both	57.7	97/168
Paracentesis of anterior chamber		
Sedation	66.3	63/95
General anaesthesia	4.2	4/95

Both	29.5	28/95
Paracentesis of vitreous		
Sedation	57.4	39/68
General anaesthesia	16.2	11/68
Both	26.5	18/68
Cataract surgery		
Sedation	0.0	0/48
General anaesthesia	97.9	47/48
Both	2.1	1/48
Vitrectomy		
Sedation	4.2	1/24
General anaesthesia	91.7	22/24
Both	4.2	1/24
Clinical setting for RBA (MCQ)		
Clinic	70.3	154/219
Field	6.3	15/219
Both	22.8	50/219
Used anaesthetics (MAQ)		
Mepivacaine	67.4	151/224
Lidocaine	56.3	126/224
Bupivacaine	37.9	85/224
Ropivacaine	3.1	7/224
Procaine	0.9	2/224
Adding of adrenaline (MCQ)		
No	93.5	203/217
Yes	6.5	14/217
Used injection method (MAQ)		
Dorsal block	88.8	199/224
4-point block	34.8	78/224
Lateral block	8.9	20/224
Modified Peterson block	2.2	5/224
Preferred injection method (MCQ)		
Dorsal block	79.6	172/216
4-point block	18.5	40/216
Lateral block	0.9	2/216
Modified Peterson block	0.5	1/216
None	0.5	1/216
Has ever observed these complications during injection (MAQ)		
No	68.8	154/224
Retrobulbar haemorrhage	22.8	51/224
Oculocardiac reflex	5.4	12/224
Globe puncture	4.9	11/224
Intravascular injection	3.6	8/224
Optic nerve puncture	3.1	7/224
Intrameningeal injection	0.0	0/224
Has ever observed these complications intraoperatively (MAQ)		
No	24.6	55/224

Lack of anaesthesia	40.6	91/224
Exophthalmos	33.5	75/224
Chemosis	32.1	72/224
Surgical field compression by the local anaesthetic	17.4	39/224
Lack of akinesia	16.1	36/224
Hypersensitivity	3.1	7/224
Oculocardiac reflex	3.1	5/224
Has ever observed these complications postoperatively (MAQ)		
No	72.3	162/224
Chemosis	19.2	43/224
Exophthalmos	8.9	20/224
Keratitis	3.6	8/224
Retrobulbar abscess	3.1	7/224
Has ever experienced a failed RBA (MCQ)		
No	53.2	115/216
Yes	46.8	101/216
in < 30% of cases	72.3	73/101
in > 30% of cases	27.7	28/101
Strategies to deal with failed RBA (MAQ)		
reapplication of RBA	82.2	83/101
with same injection method	51.8	43/83
with different injection method	48.2	40/83
subcutaneous infiltration of the surgical field	62.4	63/101
Local anaesthetic technique used for enucleation in the standing sedated horse (MAQ)		
RBA	89.0	186/209
Auriculopalpebral nerve	86.1	180/209
Supraorbital nerve	81.8	171/209
Infratrochlear nerve	63.6	133/209
Lacrimal nerve	59.3	124/209
Zygomatic nerve	57.9	121/209
Lower lid infiltration	57.4	120/209
Upper lid infiltration	50.7	106/209
Local anaesthetic on cornea	41.6	87/209

Abbreviations: MAQ, multiple answer question, more than one answer may be selected; MCQ, multiple choice question, only one answer can be selected; RBA, retrobulbar anaesthesia.
