# Supplementary material for: Global survey on the utilisation and experiences with different retrobulbar anaesthesia techniques in horses
Source: Equine Vet J. 2025 Aug 23;58(4):1091–102. doi: 10.1111/evj.70082 (PMC13244178; doi:10.1111/evj.70082)
Supplement: Supplementary file 3 — Table S2: Countries where respondents were employed at the time of completing the survey. [file EVJ-58-1091-s003.pdf]

Equine Veterinary Journal BEVA

Table S2: Countries where respondents (238 equine clinicians) of an online survey are currently employed. Data are presented as numbers (n) and percentages (%). Multiple choice question, only one answer can be selected.

Country	%	n
Germany	22.3	53
United States	20.6	49
United Kingdom	14.7	35
Belgium	5.9	14
Switzerland	5.0	12
France	4.2	10
Spain	3.8	9
Australia	2.5	6
Canada	2.5	6
Austria	2.1	5
Ireland	2.1	5
Italy	2.1	5
Netherlands	2.1	5
Sweden	2.1	5
Finland	1.7	4
Israel	1.3	3
Denmark	0.8	2
New Zealand	0.8	2
South Africa	0.8	2
Bangladesh	0.4	1
Greece	0.4	1
Mexico	0.4	1
Portugal	0.4	1
Saudi Arabia	0.4	1
Slovenia	0.4	1
