# Supplementary material for: Global survey on the utilisation and experiences with different retrobulbar anaesthesia techniques in horses
Source: Equine Vet J. 2025 Aug 23;58(4):1091–102. doi: 10.1111/evj.70082 (PMC13244178; doi:10.1111/evj.70082)
Supplement: Supplementary file 4 — Table S3: Mixture of anaesthetics drugs used by respondents. [file EVJ-58-1091-s002.pdf]

Equine Veterinary Journal BEVA

Table S3: Mixtures of anaesthetic drugs participants (N=45) used, presented as absolute numbers (n) and percentages (%), based on an online survey of equine veterinarians (N=238, multiple choice question).

Drug	Ratio	n	%
**L:B**	1:1	23	51.1
**L:B**	1:2	6	13.3
**L:M**	1:1	6	13.3
**B:M**	1:2	6	13.3
**L:M**	1:2	3	6.7

Abbreviations: L, lidocaine; B, bupivacaine; M, mepivacaine.
