# Supplementary material for: Global survey on the utilisation and experiences with different retrobulbar anaesthesia techniques in horses
Source: Equine Vet J. 2025 Aug 23;58(4):1091–102. doi: 10.1111/evj.70082 (PMC13244178; doi:10.1111/evj.70082)
Supplement: Supplementary file 5 — Table S4: Details on needle size used by respondents. [file EVJ-58-1091-s005.pdf]

Equine Veterinary Journal BEVA

**Table S4:** Details on the needle size used for retrobulbar anaesthesia in equine patients, grouped by injection technique and presented as percentages (%), based on an online survey of equine veterinarians (N=238, multiple answer question, more than one answer may be selected).

| Injection technique | N | 18G | 19G | 20G | 21G | 22G | no answer |
|---|---|---|---|---|---|---|---|
| Dorsal block | 199 | 13.6% | 7.0% | 49.7% | 11.1% | 15.6% | 6.5% |
| 4-point block | 78 | 17.9% | 7.7% | 46.2% | 16.7% | 15.4% | 3.8% |
| Lateral block | 20 | 25.0% | 0.0% | 35.0% | 10.0% | 15.0% | 15.0% |
| Modified Peterson block | 5 | 0.0% | 0.0% | 80.0% | 0.0% | 0.0% | 20.0% |
