# Supplementary material for: Global survey on the utilisation and experiences with different retrobulbar anaesthesia techniques in horses
Source: Equine Vet J. 2025 Aug 23;58(4):1091–102. doi: 10.1111/evj.70082 (PMC13244178; doi:10.1111/evj.70082)
Supplement: Supplementary file 6 — Table S5: Details on needle length used by respondents. [file EVJ-58-1091-s001.pdf]

Table S5: Details on needle lengths in millimetre (mm) used for retrobulbar anaesthesia in equine patients, grouped by injection technique and presented as percentages (%), based on an online survey of equine veterinarians (N=238, multiple answer question, more than one answer may be selected).

Injection technique	N	90mm	75mm	63mm	no answer
Dorsal block	199	48.2%	27.6%	13.6%	10.4%
4-point block	78	33.3%	32.1%	26.9%	7.6%
Lateral block	20	40.0%	25.0%	5.0%	30.0%
Modified Peterson block	5	40.0%	40.0%	0.0%	20.0%
