# Supplementary material for: Global survey on the utilisation and experiences with different retrobulbar anaesthesia techniques in horses
Source: Equine Vet J. 2025 Aug 23;58(4):1091–102. doi: 10.1111/evj.70082 (PMC13244178; doi:10.1111/evj.70082)
Supplement: Supplementary file 7 — Table S6: Volume of local anaesthetic used by respondents. [file EVJ-58-1091-s006.pdf]

Equine Veterinary Journal BEVA

Table S6: Volume of local anaesthetic in millilitre used for retrobulbar anaesthesia, grouped by injection technique, based on an online survey of equine veterinarians (N=238, multiple answer question, more than one answer may be selected).

Injection technique	N	Median	Minimum	Maximum	Range	IQR 25	IQR 75
Dorsal block	192	10.0	2.0	30.0	28.0	10.0	12.0
4-point block	66	20.0	2.0	80.0	78.0	13.75	32.5
Lateral block	18	15.0	2.0	20.0	18.0	5.0	16.25
Modified Peterson block	4	15.0	5.0	15.0	10.0	7.5	15.0

Abbreviations: IQR, inter quartile range.
