# Supplementary material for: Global survey on the utilisation and experiences with different retrobulbar anaesthesia techniques in horses
Source: Equine Vet J. 2025 Aug 23;58(4):1091–102. doi: 10.1111/evj.70082 (PMC13244178; doi:10.1111/evj.70082)
Supplement: Supplementary file 8 — Table S7: Local anaesthetic approach used for enucleation in standing horses by 23 participants who did not use retrobulbar anaesthesia. [file EVJ-58-1091-s008.pdf]

Equine Veterinary Journal BEVA

Table S7: Local anaesthetic approach used for enucleation in standing sedated horses by the 23 participants who did not use retrobulbar anaesthesia. The number of respondents (n) is given for each approach. Multiple answer question, more than one answer may be selected.

n	None	Supraorbital nerve	Infratrochlear nerve	Lacrimal nerve	Zygomatic nerve	Auriculopalpebral nerve	Infiltration lower lid	Infiltration upper lid	Local anaesthetic on cornea
1	x								
1							x	x	
2						x			
1						x		x	
5		x	x	x	x	x			
2		x	x	x	x				
1		x	x	x	x		x	x	
1		x	x	x	x	x	x	x	
1		x	x	x	x	x	x	x	x
2		x		x	x	x	x	x	
1		x	x	x		x	x	x	x
1		x					x	x	x
1		x				x	x	x	
1		x	x			x	x		
1		x	x			x			x
1		x				x	x		
